# Adult-Onset Neuronal Intranuclear Inclusion Disease with Mitochondrial Encephalomyopathy, Lactic Acidosis, and Stroke-Like (MELAS-like) Episode: A Case Report and Review of Literature

**DOI:** 10.3390/brainsci12101377

**Published:** 2022-10-11

**Authors:** Qian Zhou, Meiqun Tian, Huan Yang, Yue-Bei Luo

**Affiliations:** 1 Department of Neurology, Xiangya Hospital, Central South University, Changsha 410008, China; 2 Department of Neurology, Hua Yuan People’s Hospital, Jishou 416000, China

**Keywords:** neuronal intranuclear inclusion disease, NIID, mitochondrial dysfunction, MELAS, reversible DWI hyperintensities

## Abstract

Neuronal intranuclear inclusion disease (NIID) is a rare neurodegenerative disease with highly heterogeneous manifestations. Curvilinear hyperintensity along the corticomedullary junction on diffusion-weighted images (DWI) is a vital clue for diagnosing NIID. DWI hyperintensity tends to show an anterior-to-posterior propagation pattern as the disease progresses. The rare cases of its disappearance may lead to misdiagnosis. Here, we reported a NIID patient with mitochondrial encephalomyopathy, lactic acidosis and stroke-like (MELAS-like) episode, and reversible DWI hyperintensities. A review of the literature on NIID with MELAS-like episodes was conducted. A 69-year-old woman stated to our clinics for recurrent nausea/vomiting, mixed aphasia, altered mental status, and muscle weakness for 2 weeks. Neurological examination showed impaired mental attention and reaction capacity, miosis, mixed aphasia, decreased muscle strength in limbs, and reduced tendon reflex. Blood tests were unremarkable. The serological examination was positive for antibody against dipeptidyl-peptidase-like protein 6 (DPPX) (1:32). Brain magnetic resonance imaging (MRI) revealed hyperintensities in the left temporal occipitoparietal lobe on DWI and correspondingly elevated lactate peak in the identified restricted diffusion area on magnetic resonance spectroscopy, mimicking the image of MELAS. Skin biopsy and genetic testing confirmed the diagnosis of NIID. Pulse intravenous methylprednisolone and oral prednisolone were administered, ameliorating her condition with improved neuroimages. This case highlights the importance of distinguishing NIID and MELAS, and reversible DWI hyperintensities can be seen in NIID.

## 1. Introduction

Neuronal intranuclear inclusion disease (NIID) is a heterogeneous neurodegenerative disease, characterized by eosinophilic hyaline nuclear inclusions that are positive for ubiquitin and related proteins [1]. This disease can be sporadic or of an autosomal dominant inheritance. Recent research has identified the trinucleotide GGC repeat expansions in the 5′ untranslated region (5′ UTR) of the NOTCH2NLC gene in NIID [2,3].

Recently, Chinese researchers divided NIID into four subgroups based on the patients’ initial and main clinical manifestations: dementia-dominant, movement disorder-dominant, muscle weakness-dominant, and paroxysmal symptom-dominant types [4]. Previous cases of paroxysmal symptoms have reported encephalitic episodes, stroke-like episodes, chronic headache, and mitochondrial encephalomyopathy, lactic acidosis, and stroke-like (MELAS-like) episode [4,5,6,7]. Due to its low incidence and high heterogeneity, diagnosis of NIID is often delayed or missed in clinical practice.

Fortunately, Sone in 2016 proposed diagnostic criteria for NIID, which mainly include positive skin biopsy, genetic examination, and typical magnetic resonance imaging (MRI) findings [1]. Typical diffusion-weighted imaging (DWI) subcortical lace sign is considered an imaging marker for NIID with high specificity and sensitivity [8]. It tends to show an anterior-to-posterior propagation pattern. However, such lesions are more frequent in patients with dementia and paroxysmal symptoms types than in those with muscle weakness and movement disorder types [4]. It can also be absent in 10–20% of NIID patients, especially in the early disease course [8,9,10]. Reversible DWI hyperintensities have been reported in two cases in which hyperintense signals disappeared despite disease progression [11,12].

Reversible DWI hyperintensities can also present in MELAS syndrome, mostly caused by m.3243 A > G mutation in the MT-TL1 gene encoding the mitochondrial tRNA^Leu^ (UUR) [13]. It is a multi-organ disease with broad manifestations such as stroke-like episodes, dementia, epilepsy, lactic acidemia, myopathy, recurrent headaches, hearing impairment, diabetes, and short stature [13]. Patients tend to present stroke-like lesions inconsistent with vascular territory. These lesions with spontaneous reversibility are common in the posterior brain regions (i.e., the temporoparietal junction and the parietal and occipital lobes) [14]. Interestingly, patients with NIID can also present the above manifestations in MELAS syndrome.

Hitherto, MELAS-like episodes were only identified in two cases of NIID, among which one patient complained about chronic polyneuropathy and the other suffered recurrent migraine-attack [5,15]. Interestingly, reversible DWI hyperintensities were only seen in the case of polyneuropathy [5]. The other case presented progressed DWI hyperintensities [15]. Noticeably, several studies have also reported NIID patients with MELAS-like neuroimages, such as stroke-like lesions in the posterior brain regions (summarized in Table 1). However, the dynamics of these lesions are rarely observed.

Here, we report a sporadic NIID case mimicking MELAS episode, with detailed chronological neuroimaging. Different from the semeiology in previous cases, our patient presented recurrent nausea/vomiting, mixed aphasia, altered mental status, muscle weakness, and reversible DWI hyperintensities. A literature review is also conducted to summarize the characteristics of NIID patients with MELAS-like neuroimages, and thus better differentiate NIID and MELAS syndrome.

## 2. Materials and Methods

Clinical, imaging, and pathologic data were obtained from the patient’s case files and clinical notes. Written informed consent was also obtained.

A review of the literature was conducted on PubMed and Web of Sciences (WOS), using the following research terms-” (NIID) OR (neuronal intranuclear inclusion disease)” and the features of MELAS neuroimage including” (MELAS) OR (occipital) OR (temporal) OR (cerebral edema) OR (leukoencephalopathy) OR (hyperperfusion) OR (cortical enhancement)”. Fifties-six articles in PubMed and 64 articles in WOS have been identified. After deleting duplicates, 79 articles were scrutinized. All papers were screened by title, abstract, neuroimages, and main text. We selected papers in English reporting at least one case of NIID with MELAS-like images or episodes. Finally, eight articles have been found. For each case report/case series, the following information was attained: age, sex, NOTCH2NLC GGC repeats, initial symptoms, clinical manifestations, electroencephalograph (EEG); nerve conduction, episode, images, and treatment.

## 3. Results

The patient was a 69-year-old female farmer presenting with mild memory loss in the past two years, which did not hamper her social activity or farming. On 2 November 2021, she had a sudden visual hallucination, which resolved hours later. In the next few days, she developed headaches, recurrent vomiting, cognitive impairment, speech difficulty, and generalized weakness. She was admitted to the local hospital on November 6th, and her symptoms peaked three days after being hospitalized when she was completely bedridden. There were not any prodromal symptoms of fever. She had a ten-year history of diabetes and diabetic retinopathy, which were controlled by acarbose, gliclazide, and metformin.

**Figure 1:**
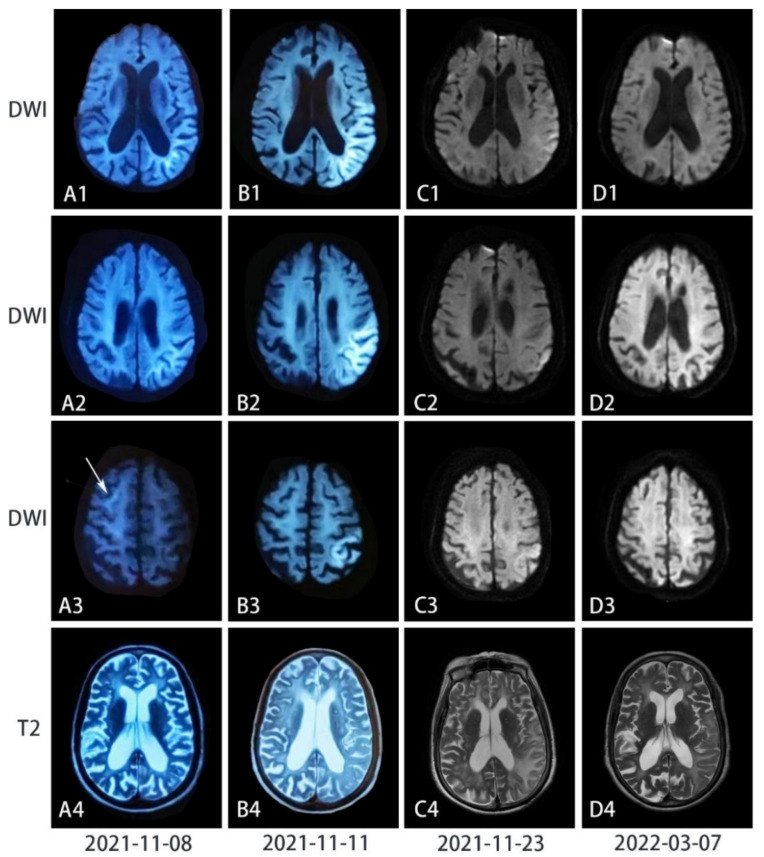
Chronological series of T2-weighted and diffusion-weighted images (DWI) images of the patient’s brain. (**A**) Seven days after the disease onset when she had cognitive impairment, mixed aphasia, and muscle weakness, DWI showed curvilinear periventricular hyperintensity, while the T2-weighted image showed diffuse brain atrophy. (**B**) Two days after her symptoms exacerbated when she could be only bedridden, curvilinear lesions had transformed into confluent high-intensity lesions on DWI along the cortex in the temporal occipitoparietal lobe (in B1), where severe encephalopathy was also observed on the T2-weighted images. However, curvilinear hyperintensity in A3 disappeared. (**C**) Half a month after the peak and the treatment of pulse intravenous methylprednisolone, most high-intensity signals on DWI were eliminated but leukoencephalopathy and cortical edema on the T2-weighted scan were exacerbated. (**D**) Four months after the symptoms onset, high-intensity signals on DWI have vanished, while hyperintensity reduced with improved brain edema on T2-weighted images.

Brain MRI in the local hospital one week after disease onset revealed symmetrical periventricular hyperintense signals on T2-weighted images, with diffuse brain atrophy and cerebral ventricle dilation (Figure 1). The lesions were unenhanced. Cerebrospinal fluid (CSF) examination indicated normal white blood cell count. Elevated protein (796 mg/L) and glucose (5.9 mmol/L) levels were observed in CSF. The testing of antibodies associated with autoimmune encephalitis and paraneoplastic syndrome revealed positive antibody against dipeptidyl-peptidase-like protein 6 (DPPX, titer 1:32) in the blood, while it was not found in CSF. Encephalitis was initially suspected. A follow-up MRI on November 11th found restricted diffusion in the left temporal occipitoparietal corticomedullary junction on DWI images and correspondingly elevated lactate peak on magnetic resonance spectroscopy (MRS), suggesting the possibility of MELAS. Pulse intravenous methylprednisolone (1 g) was given daily for three days and gradually tapered and replaced with oral prednisone. Her vomiting was alleviated, and muscle strength gradually improved. Cognitive disturbance was nevertheless persistent. She was referred to our hospital on 22 November.

Upon admission to our hospital, physical examination revealed normal vital signs. Neurological examination showed impaired mental attention and reaction capacity, miosis, mixed aphasia, decreased muscle strength in limbs generalized with MRC grading 4, and reduced tendon reflex. She could not cooperate with the examination of the muscle tone, brain nerve, ataxia, and sensory system. By then, she was unable to complete the mini-mental state examination (MMSE) scale and the Montreal cognitive assessment scale (MoCA).

Laboratory investigation revealed an elevated neutrophil percentage of 88.1 with a normal cell count. Blood glucose and glycated hemoglobin levels were elevated (11.43 mmol/L and 7.4%, respectively). Her serum creatine kinase, lactic acid, ketone, homocysteine, immunoglobulin, sex hormone, and cortisol level were all normal.

**Figure 2:**
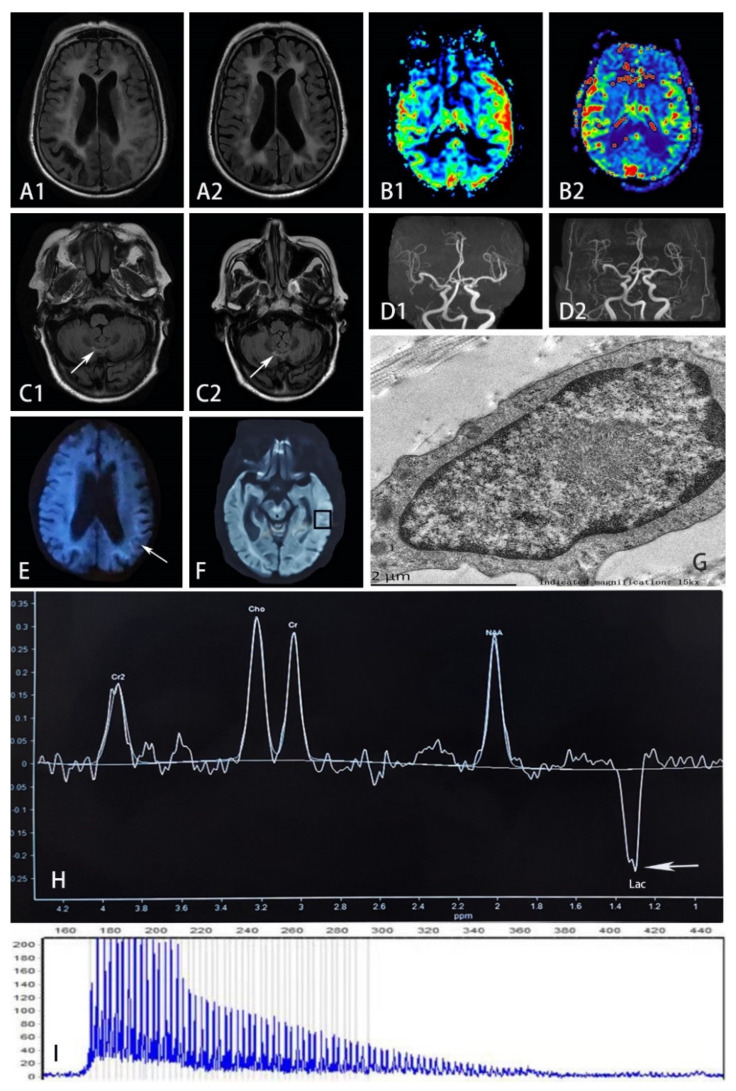
(**A**) FLAIR sequence on November 23rd (in A1) showed hyperintensity and edematous areas in the left temporal and occipital lobes, while such lesions improved 4 months later with reduced hyperintensity and less edema shown in A2. (**B**) PWI on November 23rd (in B1) showed prominent hyperperfusion in accordance with the lesion, but such phenomenon did not present after 4 months with symmetrical blood flow in bilateral temporal lobes shown in B2. (**C**) FLAIR sequence on November 23rd showed hyperintensity on the paravermis area (in C1), which remained the same after 4 months shown in C2. (**D**) MRA on November 23rd (in D1) showed the stenosis of M2 segments of the right middle cerebral artery, which was also presented after 4 months in D2. (**E**) The arrow refers to high-intensity areas in the corticomedullary junction on DWI on November 8th. (**G**) Electron microscopy of mechanocyte showed dense filamentous materials without limiting membrane. (**H**) MRS focusing on the temporal lesion (in (**F**)) showed the lactate peak appearance (shown by arrow). (**I**) RP-PCR showed GGC expansion in the patient. (FLAIR, fluid-attenuated inversion recovery; PWI, perfusion-weighted image; DWI, diffusion-weighted image; MRA, magnetic resonance angiography; MRS, magnetic resonance spectroscopy; RP-PCR, repeat-primed PCR; Lac, lactate).

Electroencephalogram demonstrated diffuse slow waves (1.5–7 c/s) on the left hemisphere and limited sharp waves, especially on the frontotemporal lobe. Nerve conduction study was consistent with demyelinating polyneuropathy predominantly involving the motor nerves. The patient’s bilateral median (left, 35.2 m/s and right, 42.3 m/s) and ulnar nerves (38.8 m/s on both sides) showed slow velocity. Motor conduction velocity of her bilateral tibial and peroneal nerves was also reduced to 30 m/s on average. Gynecological Sonography was normal. Repeated MRI on November 23rd showed restricted diffusion signals along the temporal occipitoparietal juxtacortex, and stenosis of M2 segments of the right middle cerebral artery and bilateral posterior cerebral arteries. Perfusion-weighted imaging (PWI) showed prominent hyperperfusion in the left occipitotemporal cortex (Figure 2). Faint DWI hyperintensity in the corticomedullary junction and high-intensity signals in the paravermis area were retrospectively recognized, suggesting the possibility of NIID.

Skin biopsy samples were obtained 10 cm above the patient’s ankle. Electron microscopy showed round-shaped intranuclear inclusions, composed of dense filamentous materials without membrane structure. However, eosinophilic ubiquitin-positive and p62-positive intranuclear inclusions were not found. Repeat-primed PCR confirmed the diagnosis of adult-onset NIID (>66 repeats of GGC in the 5′UTR of the NOTCH2NLC gene). Her family history was unremarkable. Our patient and her brother were entrusted to their relatives since childhood, and they soon lost contact with their parents. Her brother with diabetes did not suffer from migraine, stroke, or deafness. Her offspring are healthy. No DNAs of other family members were available.

During hospitalization, oral prednisolone was gradually tapered. Her cognitive deficiency was gradually alleviated, and she could briefly communicate when she was discharged. After returning home, the patient had recurrent vomiting for a week but resolved spontaneously.

On follow-up three months after discharge, she had pupils of diameters within normal range. She was ambulant and alert. MMSE score was 18 and MOCA was 8, indicating moderate cognitive impairment. A follow-up brain MRI revealed the DWI high intensity of the occipitotemporal lobe had largely resolved, while brain atrophy progressed.

## 4. Discussion

Here we report a patient with NIID presenting with an acute MELAS-like episode and neuroimaging. She had insidious onset of cognitive impairment, and the disease was exacerbated by a MELAS-like episode. Brain imaging showed diffuse white matter hyperintensity, brain atrophy, and a largely reversible parietooccipital lesion. Despite the positive anti-DPPX antibody, the diagnosis of autoimmune encephalitis was dismissed as the clinical picture did not conform to the phenotype associated with anti-DPPX encephalitis [19]. Lactate peak on MRS and hyperperfusion in the lesion have been reported in both MELAS and NIID [5]. Nevertheless, no muscle volume reduction or myogenic changes on electromyography (EMG) have been detected. The late onset age and lack of any family history of muscle weakness or stroke-like episodes made the diagnosis of mitochondrial disease very unlikely. The faint corticomedullary lesion on DWI highly suggestive of NIID resolved as the MELAS-like episode ended, but hyperintensity of the paravermis area persisted. Such neuroimages suggested the possibility of NIID.

Corticomedullary hyperintensity on DWI is a strong image marker for NIID with an anterior-to-posterior propagation pattern [8]. Previous literature has also identified high DWI signals in the corpus callosum, severe leukoencephalopathy involving the corpus callosum, middle cerebellar peduncle, paravermis area, and cortical edema with gadolinium enhancement as useful clues. Cerebral atrophy and lateral ventricle enlargement were often observed in the late stages [4].

Interestingly, Sone reported that 21% of adult-onset NIIDs experienced a subacute encephalitic episode with characteristic symptoms including fever, headache, vomiting, and loss of consciousness [1]. NIID patients with encephalopathy tend to have cortical hyperintensity distributed in the parietal-occipital lobes, in contrast to curvilinear DWI hyperintensity preferentially located in the frontoparietal region [8]. These lesions are not distributed following vascular supply [8]. One-fifth of encephalitis-like NIID cases could have FLAIR hyperintense lesions with edematous changes and contrast enhancement [8]. Okubo reported a NIID patient with focal hypoperfusion at the acute stage of encephalitis-like episode and rebound hyperperfusion several days later [16]. Ataka and Ishihara reported hyperperfusion in the abnormal cortices [5,20]. The increased peak of lactate point on MRS can also be observed in NIID [5].

Similar to the encephalopathy of NIID, cortical swelling and hyperperfusion can also present in MELAS. Therefore, it can easily be confused with the neuroimaging of MELAS.

To summarize the characteristics of NIID with MELAS-like neuroimaging, we reviewed the literature and found another 11 cases with MELAS-like neuroimages (Table 1). All patients with MELAS-like neuroimages experienced encephalitis-like episodes. A third of the patients used to have migraine. They tend to present headaches (83.3%), altered mental status (66.7%), memory decline (58.3%), and nausea/vomiting (50.0%) during an episode. All patients who received examination of electroencephalograph presented slow waves. Myelin damages (60%) were more frequent than axonal damages (20%). All patients have brain edema and cortical lesions. Cortical enhancement and DWI corticomedullary hyperintensities have been shown in most cases.

Interestingly, the aforementioned manifestations can also be present in MELAS syndrome, except corticomedullary hyperintensities on DWI. Thus, curvilinear hyperintensity along the corticomedullary junction on DWI is a useful tool for differential diagnosis.

Unlike previous cases with cortical enhancement, which is purposed as a brain image marker for the differential diagnosis between MELAS and NIID with MELAS-like episodes, our patient did not present such enhancement. In fact, such enhancement can also be seen in MELAS due to hyperemia or luxury perfusion [13]. Furthermore, our case showed the disappearance of DWI hyperintensities as the episode ended, reversed to the common idea that this signal would not fade away once appeared. Skin biopsy, with a high degree of consistency with NOTCH2NLC gene detection, can reveal eosinophilic intranuclear inclusions, immunopositive for ubiquitin and p62, composed of fibrous materials without membranous structures [21]. However, fibrous materials without membranes were identified in mechanocyte nucleus under electron microscope, while immunofluorescence staining was negative in our patient.

Eosinophilic intranuclear inclusions are valuable diagnostic clues. It can be detected more than 10 years before the onset of the symptoms and found in morphologically intact neurons without obvious neuronal loss. Previous literature demonstrated the positive rate of electron microscopy was lower than that of immunostaining due to limitations in the sampling and observation scope [21,22]. However, it is exactly opposite in our patient. Cao also reported two patients with negative pathological findings [22]. Though the underlying mechanism is unclear, the loss of p62/SQSTM1 has been reportedly associated with accelerating aging and age-related pathologies [23]. Moreover, eosinophilic ubiquitin-positive inclusions can also be observed in fragile X-associated tremor/ataxia syndrome (FXTAS) in neurons, glial cells, and somatic cells, though no skin pathological or electron microscopic findings of intranuclear inclusions have been identified in FXTAS [1]. NIID and FXTAS shared similar clinical manifestations. Thus, it is difficult to distinguish NIID from FXTAS with only the histopathological findings. For this reason, gene tests should be performed. NIID is diagnosed based on abnormal repeat expansion of GGC (>65 repeats) within the 5′ UTR of the NOTCH2NLC gene [4].

Though curvilinear hyperintensity along the corticomedullary junction is a useful tool to differentiate MELAS and NIID with MELAS episode, it can be absent or reversible in rare cases. Some researchers observed such hyperintensities were absent at the onset but presented in the corticomedullary junction area 6 years later, and ultimately disappeared 8 years after onset in a NIID patient [24]. Researchers suspected the pathological spongiotic changes in subcortical white matter proximal to U-fibers may be the culprit of DWI hyperintensities [11,25]. Subsequent edema withdrawal, neuronal loss, and gliosis may account for the disappearance of DWI hyperintensities and the widening of cerebral sulci [11]. Moreover, patients with MELAS can present spontaneous reversibility in both neurological symptoms and neuroimages [14]. Once such curvilinear hyperintensities disappear, it is easily misdiagnosed.

To distinguish between NIID with MELAS-like episodes and MELAS syndrome, we should consider the following conditions. For clinical features, most patients with confirmed MELAS have a maternal genetic history and present between 2 and 40 years of age, mostly before age 15. They had systemic symptoms including loss of hearing, growth failure, and diabetes, which are distinguished from phenotypes of NIID [14]. For auxiliary examination, an electrophysiological study might aid the differentiation. Nerve conduction studies of MELAS patients typically show an axonal or mixed axonal and demyelinating neuropathy, similar to NIID in which demyelination is more frequent than axonal damage [1,13]. EMGs of MELAS patients tend to show myogenic changes. In addition to corticomedullary hyperintensities on DWI, basal ganglia calcification and iron deposition can be frequently seen in MELAS, while they can also be presented in the elders [13]. High intensity in the paravermis area might be a useful indicator to distinguish NIID as it is rarely reported in other patients with leukoencephalopathies [4]. Muscle and skin biopsy, as well as gene testing aid the confirmative diagnosis.

Bearing many commonalities in clinical and imaging characteristics, NIID is suspected to have similar pathogenesis to MELAS. Noticeably, previous researchers also identified abnormal mitochondrial inclusions in patients with NIID [26]. Yu et al., recently developed a fly model of CGG repeat expansion in NOTCH2NLC, recapitulated key pathological and clinical features of NIID, and characterized the mitochondrial dysfunction in these model organisms, human samples, and cellular models [27]. Here, we proposed three possible mechanisms linking NIID to mitochondrial dysfunction. First, large CGG repeats may stall the replication fork leading to a double-strand break and chromosome fragility, which further affects mitochondrial homeostasis [28]. Second, expanded CGG repeats may sequester specific RNA binding proteins resulting in altered splicing and decreased miRNA biogenesis. Decreased expression of miRNAs leads to their altered translocation to the mitochondria [29]. Lastly, polyG forms intranuclear inclusion may affect the transport of miRNA/mRNAs and nuclear-encoded mitochondrial proteins to the mitochondria. These effects can finally result in bioenergetic crisis, elevated reactive oxygen species, and cell death [29]. The secondary mitochondrial abnormalities may at least partly explain the overlapping presentations and neuroimages of NIID and MELAS.

## 5. Conclusions

This case report portrays a patient with NIID who presented MELAS-like episode and reversible DWI hyperintensities. Patients with MELAS-like neuroimaging tend to present headaches, altered mental status, memory decline, and nausea/vomiting during an episode. DWI curvilinear hyperintensity could aid the differentiation between NIID and MELAS, but its disappearance might lead to misdiagnosis. To distinguish between NIID with MELAS-like episodes and MELAS syndrome, we should consider some special clinical manifestations, EMG, and neuroimaging. High DWI signals in the corpus callosum and severe leukoencephalopathy involving the corpus callosum, middle cerebellar peduncle, and paravermis area can help the diagnosis of NIID. Muscle and skin biopsy, as well as gene testing aid the confirmative diagnosis.

## Figures and Tables

**Table 1:** The clinical characteristics of NIID patients with MELAS-like neuroimages.

References	Patient No.	Age, Sex	NOTCH2NLC GGC Repeats	Initial Symptoms	Clinical Manifestations	EEG	Nerve Conduction	Encephalitic Episode	FLAIR	T1W1+C	DWI Hyperintense Lesions		Treatment
									Brain Edema	Cortical Enhancement	Cortical Lesions	Corticomedullary Junction Lesions	
Our case	1	F/69	#	Amnesia	Headache; N/V; AMS; memory decline	LH diffuse SW	MD of motor nerve	+	+	-	+	+	Methylprednisolone
Xie [6]	2	F/51	118	Migraine	Headache; SD; urinary retention	N.A.	N.A.	+	+	+	+	+	Coenzyme Q10, riboflavin
Wang [7]	3	M/20	N.A.	Migraine	Headache; muscle weakness; SD; seizures; memory decline	N.A.	MD of motor nerve	+	+	-	+	+	Anti-seizure medications;
Okubo [16]	4	M/50	143	Tremor	Memory decline; tremor	N.A.	#	+	+	N.A.	+	+	N.A.
Ishihara [5]	5	F/47	*	Muscle weakness; SD	Headache; N/V; AMS	N.A.	#	+	+	+	+	-	Anti-seizure medications; edaravone, taurine
Liang [17]	6	F/56	115	Migraine	Headache; aphasia; fever; N/V; muscle weakness; SD; AMS; memory decline	RH diffuse SW	Normal	+	+	+	+	-	Methylprednisolone, dehydration
	7	F/35	98	migraine	Headache; N/V; muscle weakness; AMS; memory decline	BH sporadic SW	N.A.	+	+	+	+	+	Methylprednisolone, dehydration
	8	M/56	123	Dysuria; amnesia	Headache; bladder dysfunction; N/V; memory decline; AMS; SD	BH diffuse SW	AD of motor and sensory nerve	+	+	+	+	+	Methylprednisolone, dehydration
	9	F/61	110	Tremor	Headache; memory decline; bladder dysfunction; AMS; SD	BH diffuse SW and sporadic SSW	MD of sensory nerve	+	+	+	+	+	Methylprednisolone, dehydration
Liu [8]	10	F/48	136	N.A.	Headache; seizures; N/V	N.A.	N.A.	+	+	+	+	+	N.A.
	11	F/79	73	N.A.	AMS; seizures; aphasia; SD	N.A.	N.A.	+	+	+	+	+	N.A.
Mori [18]	12	F/61	N.A.	Parkinsonism	Headache; dysarthria; muscle weakness; AMS	N.A.	N.A.	+	+	+	+	+	Steroid therapy

No.: number; F: female; M: male; N/V: nausea/vomiting; AMS: altered mental status; EEG: electroencephalograph; DWI: Diffusion weighted imaging; FLAIR: Fluid-attenuated inversion recovery images; T1W1+C: T1 weighted images with gadolinium enhancement; Rt: right; Lt: left; RH: right hemisphere; LH: left hemisphere; BH: bilateral hemisphere; SD, sensory disturbance; MD: myelin damage; AD: axonal damage; SW: slow waves; SSW: spine-slow integrated waves; N.A.: not available; -: negative; #: positive but without details; +: positive; *: (GGC)88(GGGA)1{(GGC)4(GGA)2}9(GGC)4(GGA)1(GGC)3(GGA)2(GGC)2.
